# UNDERREPRESENTED FAMILY CAREGIVERS: BUILDING TRUST TO STRENGTHEN RECRUITMENT AND RETENTION

**DOI:** 10.1093/geroni/igae098.0962

**Published:** 2024-12-31

**Authors:** Gilbert Gimm, Beth Fields

**Affiliations:** Chair; Discussant

## Abstract

The definition of “family caregiver” typically excludes many groups, including those from different genders, racial/ethnic identities, and caregiver age categories. Relationship building, establishing trust with community partners, and interdisciplinary collaborations are essential for the successful recruitment, engagement, and retention of underrepresented family caregivers in research. Moreover, inclusive communication is critical to building trust and strengthening participant engagement. Each individual presentation of the symposium will focus on effective strategies to engage underrepresented family caregivers from a diverse range of age, gender, and racial/ethnic populations. Our first speaker (G. Gimm) will present findings on the effect of a 9-week stress management intervention on mean burden scores for 97 family caregivers, including 16 male caregivers. Our second speaker (K. Moss) will describe effective recruitment and retention strategies for feasibility and acceptability testing of a co-created virtual peer support intervention (Pair 2 Care), which was designed for and with Black/African American dementia family caregivers. Our third speaker (A. Kalvesmaki) will describe what is known about caregiving across the lifespan, including youth caregivers who are less than 18 years, and identify barriers to service use, as well as effective strategies to build trust in family caregiving communities. Our fourth speaker (M. Kavanaugh) will share key findings on the effective engagement of multidisciplinary healthcare providers who received training to deliver the YCare program, a hands-on caregiving skills session empowering youth caregivers. Our discussant (B. Fields) will then summarize cross-cutting themes and share practical and policy implications for researchers and underrepresented family caregivers across the lifespan.

